# The Role of *Yersinia enterocolitica* O:3 Lipopolysaccharide in Collagen-Induced Arthritis

**DOI:** 10.1155/2020/7439506

**Published:** 2020-11-12

**Authors:** Katarzyna Kasperkiewicz, Anna S. Świerzko, Marta Przybyła, Janusz Szemraj, Jarosław Barski, Mikael Skurnik, Andrzej Kałużyński, Maciej Cedzyński

**Affiliations:** ^1^University of Silesia in Katowice, Faculty of Natural Sciences, Institute of Biology, Biotechnology and Environmental Protection, Jagiellonska 28, 40-032 Katowice, Poland; ^2^Laboratory of Immunobiology of Infections, Institute of Medical Biology, Polish Academy of Sciences, Lodowa 106, 93-232 Lodz, Poland; ^3^Department for Experimental Medicine, Medical University of Silesia, Medyków 4, 40-752 Katowice, Poland; ^4^Department of Medical Biochemistry, Medical University of Lodz, Mazowiecka 6/8, 92-215 Lodz, Poland; ^5^Department of Bacteriology and Immunology, Medicum, Human Microbiome Research Program, Faculty of Medicine, University of Helsinki, Haartmaninkatu 3, 00290 Helsinki, Finland; ^6^Division of Clinical Microbiology, HUSLAB, University of Helsinki and Helsinki University Hospital, 00290 Helsinki, Finland; ^7^Department of Clinical Pathomorphology, Polish Mother's Memorial Hospital Research Institute, Rzgowska 281/289, 93-338 Lodz, Poland

## Abstract

*Yersinia enterocolitica* O:3 is mentioned among the most common arthritogenic pathogens. Bacterial components (including lipopolysaccharide (LPS)) may persist in the joint after eradication of infection. Having an adjuvant activity, LPS may enhance production of anticollagen antibodies, involved in the pathogenesis of rheumatoid arthritis. Furthermore, its ability to activate complement contributes to the inflammation. The aim of this work was to investigate whether *Yersinia* LPS (coinjected with collagen) is associated with arthritis progression or other pathological effects and to elucidate the mechanism of this association. It was demonstrated that murine mannose-binding lectin C (MBL-C) recognizes the inner core heptoses of the Rd1 chemotype LPS of *Yersinia.* In addition, the Rd1 LPS activates the MBL-associated serine protease 1 (MASP-1) stronger than the S and Ra chemotype LPS and comparable to *Klebsiella pneumoniae* O:3 LPS. However, in contrast to the latter, *Yersinia* Rd1 LPS was associated neither with the adjuvancity nor with the enhancement of pathological changes in animal paws/impairment of motility. On the other hand, it seemed to be more hepatotoxic when compared with the other tested endotoxins, while the enlargement of inguinal lymph nodes and drop in hepatic MBL-C expression (at the mRNA level) were independent of LPS chemotype. Our data did not suggest no greater impact *Y*. *enterocolitica* O:3 on the development or severity of arthropathy related to anticollagen antibody-induced arthritis in mice, although its interaction with MBL-C and subsequent complement activation may contribute to some adverse effects.

## 1. Introduction

Reactive arthritis (ReA), is a self-limiting disease; however, even 60% of patients may develop chronic arthritis [1]. The development of ReA is considered to be associated with past genitourinary or gastrointestinal infections [2]. *Yersinia enterocolitica* O:3 (YeO3) is mentioned among the most common arthritogenic agents [3]. Bacteria may gain access to the circulation and then be transferred to the joint either *via* plasma or within lymphatic cells [4]. It was suggested that, after eradication of bacteria, their cellular components (including lipopolysaccharide (LPS, endotoxin)) may still persist in the joint and contribute to pathological effects [4]. Interestingly, the presence of *Yersinia* antigens (including LPS) was demonstrated in synovial fluid cells [5]. A strong arthritogenic potential of *Y*. *enterocolitica* O:8 LPS was demonstrated by Di Genaro et al. [6] in a hamster model. Moreover, Granfors et al. [7] detected *Yersinia* LPS in leukocytes of patients with *Y*. *enterocolitica* O:3-triggered ReA even four years after infection. Interestingly, Wuorela et al. [8] showed that in the result of intracellular processing, the majority of LPS molecules presented on the surface of monocytes are devoid of the O-polysaccharide region.

Autoimmunity to collagen is suggested to be involved in the pathogenesis of rheumatoid arthritis (RA). The animal model used often to study rheumatoid arthritis is collagen-induced arthritis (CIA) following immunization with collagen type II (CII) accompanied with an adjuvant and leading to synovitis, pannus formation, erosion of bone and cartilage, and fibrosis [9]. The complete Freund's adjuvant is a commonly used agent. It is believed to induce an overstimulation of innate immune reactivity, leading to systemic inflammatory response and contributing to the development of humoral and cellular immune response, including the production of CII-specific antibodies as well as cellular reactivity to that molecule [10]. A similar activity was demonstrated for LPS: Terato et al. [11] showed that oral coadministration of *E*. *coli* LPS and CII enhanced the antibody production and T-cell response to CII.

The adjuvant activity of LPS was shown to be generally associated with its lipid A moiety [12]. However, Kido et al. [13] described a very strong adjuvant activity for LPS carrying O-specific polysaccharides (OPS) consisting of mannose homopolymers (as is the case of *Klebsiella pneumoniae* O:3, KO3). Further, the ability of the KO3 LPS to enhance the antibody production against the thyroid, testis, salivary gland, and colon extracts was demonstrated [14–16]. Interestingly, Takahashi et al. [17] reported that repeated immunization with the mixture of porcine CII and KO3 LPS caused the joint destruction, synovial hyperplasia with proliferation of synovial cells, and infiltration of inflammatory cells.

The development of acquired immunity leading to antibody production may be associated with the activation of complement system. Dempsey et al. [18] showed that the attachment of complement C3 activation product (C3d) to hen egg lysozyme significantly enhances its immunogenicity. In the case of KO3 LPS, its strong adjuvant activity may be attributable to its interaction with mannose-binding lectin (MBL) and ability to activate complement *via* the lectin pathway. Recently, Man-Kupisińska et al. [19] demonstrated that MBL binds to the LPS O-specific polysaccharide of some and to the core oligosaccharide (both its outer (OC) and inner (IC) parts) of many enterobacterial strains.

**Figure 1:**
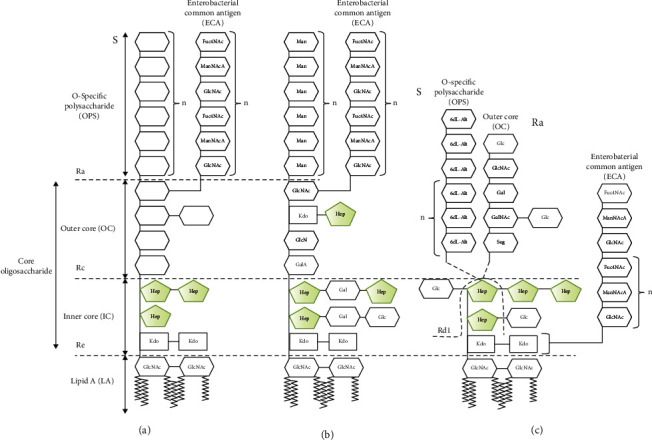
Schematic structures of *K*. *pneumoniae* O:3 and *Y*. *enterocolitica* O:3 LPS. (a) LPS typical for majority of *Enterobacteriaceae*. The empty hexagons reflect the sugar residue variability in the outer core oligosaccharide (OC) and O-specific polysaccharide (OPS) structures of different strains. The zigzag lines in LA represent fatty acids. (b) Schematic structure of *K*. *pneumoniae* O:3 LPS. (c) Schematic structures of *Y*. *enterocolitica* serotype O:3 strain and its mutants used in this work. The chemotypes of the YeO:3 LPS molecules are indicated by letter abbreviations and the dashed lines indicate the point(s) of truncations: S: LA-IC-OPS; Ra: LA-IC-OC; Rd1: LA-Kdo-Hep; Re: LA-Kdo. The wild-type O:3 bacteria produce a mixture of S- and Ra-type LPS molecules. The sugar residues: 6dL-Alt: 6-deoxy-l-altropyranose; Hep: d-glycero-d-manno-heptopyranose; Fuc4NAc: *N*-acetyl-d-fucos-4-amine (4-acetamido-4,6-dideoxy-d-galactopyranose); GalNAc: *N*-acetyl-d-galactosamine (2-acetamido-2-deoxy-d-galactopyranose); Glc: d-glucopyranose; GlcNAc: *N*-acetyl-d-glucosamine (2-acetamido-2-deoxy-d-glucopyranose); ManNAcA: *N*-acetyl-d-mannosaminouronic acid (2-acetamido-2-deoxy-d-mannopyranosuronic acid); Kdo: 3-deoxy-d-manno-oct-2-ulopyranosonic acid; l,d-Hep: l-glycero-d-manno-heptopyranose; Sug: 2-acetamido-2,6-dideoxy-d-xylo-4-ulopyranose; GalA: galacturonic acid; Gal: galactose; Man: mannose [20–24].

In contrast to common LPS structure arrangement (LA-IC-OC-OPS) (LA: lipid A; IC: inner core oligosaccharide; OC: outer core oligosaccharide; and OPS: O-specific polysaccharide) (see Figures 1(a) and 1(b)), *Y*. *enterocolitica* O:3 endotoxin is built up of lipid A and inner core oligosaccharide (LA-IC) substituted with long OPS (LA-IC-OPS) or with short OC (LA-IC-OC) (see Figure 1(c)). Moreover, in OPS-carrying molecules, IC may be additionally substituted with enterobacterial common antigen (ECA) polysaccharide (LA-IC-ECA-OPS). Previously, we demonstrated human MBL to recognize *Y*. *enterocolitica* O:3 LPS inner core heptoses [24]. That interaction resulted in complement activation *via* the lectin pathway (LP). Moreover, the substitution of the inner core with outer core oligosaccharide or O-specific polysaccharide prevented MBL binding and subsequent complement activation. Therefore, in fact, mannose-binding lectin was able to bind LPS isolated from Rc and Rd class mutants only. Moreover, MBL, as well as some other factors involved in complement lectin pathway, was detected in synovial fluid samples from patients suffering from juvenile idiopathic arthritis, another arthropathy. In sera of some of those patients, *Yersinia*-reactive antibodies were present [25]. Recently, a number of reports demonstrating the role of factors specific for complement lectin pathway in arthritis development were published [26–28]. The aim of this work was to study whether the *Yersinia enterocolitica* O:3 LPS contributes to the progression of arthritis *via* the adjuvant activity (i.e., by enhancement of synthesis of arthritogenic anti-CII antibodies) or *via* the induction of the inflammatory response through complement activation.

## 2. Materials and Methods

### 2.1. Bacterial Strains, Growth Conditions, and LPS Isolation

Bacteria used in this work are listed in Table 1. Bacteria were grown aerobically at 37°C, in LB medium in the presence of kanamycin or chloramphenicol, when required. The LPS from smooth *Y*. *enterocolitica* O:3 (6471/76-c) and *K*. *pneumoniae* O:3 strains were isolated by the hot phenol/water method according to Westphal and Jann [29]. The LPS of the rough strains (YeO3-c-R1 and YeO3-c-R1-M181) was isolated by the hot phenol/water extraction followed by the phenol/chloroform/petroleum ether method [29, 30].

### 2.2. Animals

BALB/c (H-2^d^) mice (males, 6–9 weeks old) were purchased from the animal facility of the Department for Experimental Medicine, Medical University of Silesia. Approval of the Local Ethical Commission for Animal Experimentation (number: 14/2012) (Katowice, Poland) was obtained, and the work was carried out in accordance with the National Institute of Health Guide for the Care and Use of Laboratory Animals (86/609/EEC). All mice were kept under a 12 h light/dark cycle (lights on at 7.00), a temperature of 22 ± 2°C and humidity at 50 ± 5% with free access to water and standard chow diet (Labofeed, Morawski, Poland).

### 2.3. Collagen-Induced Arthritis

To evoke collagen-induced arthritis (CIA) in BALB/c mice, the procedure described by Takahashi et al. [17] was employed. Briefly, mice (7 per group) were immunized six times subcutaneously into the inguinal region with the mixture containing 200 *μ*g of type II chicken collagen (Sigma-Aldrich, USA) and 100 *μ*g of tested LPS in a total volume of 150 *μ*l, at the intervals of 30 days. Mice immunized with CII only or injected with PBS with no antigen were used as controls. Twenty days after last immunization, mice were weighted, tested in CatWalk gait analysis system (see below), and sacrificed. Blood for serum isolation, lymph nodes, and livers were isolated. All possible steps were taken to avoid animal suffering at each stage of the experiment.

### 2.4. Assessment of Arthritis

To evaluate disease activity, the video-based CatWalk gait analysis system was employed according to the method described by Masocha and Parvathy [32]. Briefly, each mouse was placed individually in the CatWalk XT 9.1 (NOLDUS Information Technology, Wageningen, Netherlands) walkway. The mouse was allowed to walk freely and traverse from one side to the other of the walkway glass plate. Data are presented as mean + SEM according to Parvathy and Masocha [33]. One-way ANOVA followed by Dunnett's multiple comparison test were used for the determination of statistical significance.

### 2.5. Detection of MBL-A and MBL-C in Murine Serum

To estimate the MBL-A and MBL-C level changes in the course of the collagen-LPS treatment, the NUNC Maxisorp U96 microtiter plates were coated with mannan from *Saccharomyces cerevisiae* (Sigma-Aldrich), 10 *μ*g/well. After blocking and incubation with tested sera diluted in imidazole buffer (40 mM imidazole, 1.25 M NaCl, 50 mM CaCl_2_, and pH 7.8), supplemented with 0.1% BSA, the bound proteins were detected with MBL-A- and MBL-C-specific monoclonal rat antibodies (clone 8G6 and 14D12, respectively; Hycult Biotech, Netherlands) and HRP-labeled goat anti-rat Ig (Dako, Denmark).

### 2.6. MBL-A and MBL-C Interaction with LPS

To test the interaction of murine MBLs with the different LPS, the NUNC Maxisorp U96 microtiter plates were coated with 10 *μ*g/well of LPS. After blocking and incubation with tested sera diluted in imidazole buffer, supplemented with 0.1% BSA, the bound proteins were detected as described above.

### 2.7. LPS-Induced Murine MASP-1 Activation

To determine the ability of LPS to activate MBL-associated serine protease-1 (MASP-1) in mouse serum, the method described by Presanis et al. [34] was used, with slight modification. The wells of PerkinElmer HB white plate were coated with the different LPS (5 *μ*g/100 *μ*l of PBS). After blocking with buffer containing 20 mM HEPES, 140 mM NaCl, and 5 mM EDTA (pH 7.4), the mouse sera were diluted in a serum dilution buffer (40 mM HEPES, 2 M NaCl, 10 mM CaCl_2_, and pH 7.4). After overnight incubation at 4°C and washing twice with high ionic strength wash buffer (20 mM HEPES, 1 M NaCl, and 5 mM CaCl_2_) and twice with wash buffer (20 mM HEPES, 140 mM NaCl, 5 mM CaCl_2_, and pH 7.4) heated to 37°C, the substrate for MASP-1 (0.1 mM Val-Pro-Arg-AMC (VPR-AMC, Bachem, Switzerland)) in 20 mM HEPES, 5 mM CaCl_2_ buffer (pH 8.5) was added. The samples were excited at 355 nm, and emission was read at 460 nm every 30 s for 1 h, using Varioscan Flash reader (ThermoFisher Scientific, USA). For negative control, the addition of serum was omitted.

### 2.8. Investigation of MBL-A- and MBL-C-Specific mRNA Expressions

MBL-A and MBL-C gene expressions in the liver were determined using real-time PCR method with the help of primers described by Baldo et al. [35]. Total RNA was extracted by the TRIzol reagent method (Invitrogen Life Technologies, USA) using the single-step purification protocol [36] and processed to cDNA synthesis. The MBL-A, MBL-C, and *β*-actin expressions were quantified by real-time PCR using ABI Prism 7000 sequence detection system (Applied Biosystems, USA) according to the manufacturer's protocol. Briefly, 2.5, 2.0, 1.5, 0.5, and 0.25 *μ*l of synthesized cDNA were amplified in triplicate for *β*-actin and each of the target genes to create a standard curve. Likewise, 2 *μ*l of cDNA was amplified in triplicate in all isolated samples for each primer/probe combination and *β*-actin. Each sample was supplemented with both respective 0.3 *μ*M forward and reverse primers, fluorescent probe, and made up of 50 *μ*l using qPCR™ Mastermix for SYBR green I (Eurogentec, Belgium). All samples were incubated at 50°C for 2 min and at 95°C for 10 min and then cycled at 95°C for 30 s, 55°C for 1 min, and 72°C for 1 min (40 cycles). SYBR Green I fluorescence emission data were captured and mRNA levels were quantified using the critical threshold (Ct) value. Data were analyzed with the help of ABI Prism 7000 SDS software. Controls without RT and with no templated cDNA were performed with each assay. To compensate for variations in input RNA amounts and efficiency of reverse transcription, *β*-actin mRNA was quantified and results were normalized to these values. Relative gene expression levels were obtained as *ΔΔ*Ct [37]. Specificity of amplification was further confirmed by obtaining melting curve profiles [37].

## 3. Results

### 3.1. Collagen Type II and *Yersinia* LPS Mixture Is Arthritogenic

To assess the possible contribution of *Yersinia* LPS to collagen-induced arthritis, BALB/c mice were immunized with type II chicken collagen (CII) mixed either with *Yersinia* wild-type LPS (6471/76-c), LPS with complete outer core (YeO3-c-R1, Ra-chemotype), or LPS reduced to the lipid A-inner core (YeO3-c-R1-M181, Rd1 chemotype), according to Takahashi et al. [17]. Mice immunized with CII mixed with *K*. *pneumoniae* O:3 LPS were used as a positive control. The obtained results were compared with those from mice treated with CII or PBS only.

**Figure 2:**
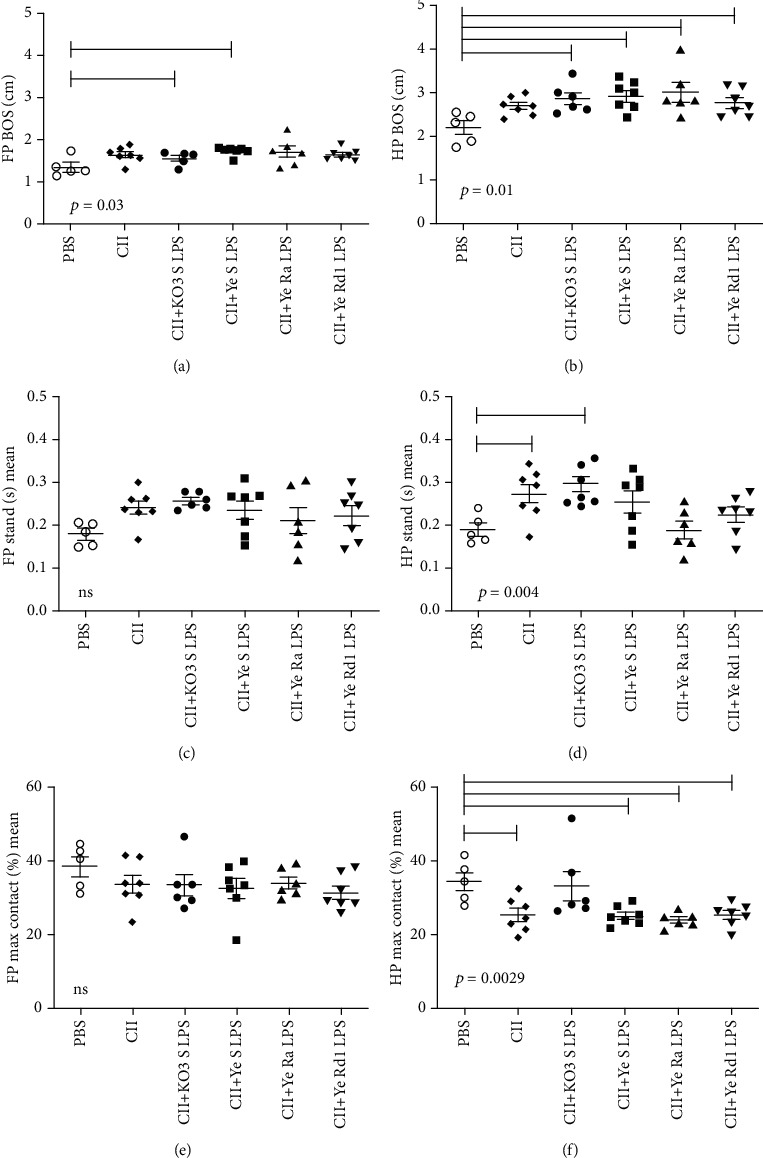
CatWalk gait analysis of mice injected with PBS, collagen type II, and the mixture of CII and tested LPS. Individual values from 5 (negative control, PBS) to 7 animals and means ± SEM are demonstrated. Statistically significant differences in comparison with baseline: *p* < 0.05 (one-way ANOVA followed by Dunnett's multiple comparison test). FP/HP span, FP/HP stand (s) mean, and FP/HP max contact (%) mean were analyzed. Base of support (BOS): the average width between either the front paws or the hind paws; Stand (s): the duration of contact with the glass plate of the print, or is the duration in seconds of contact of a paw with the glass plate; Max contact at (s): the time in seconds since the start of the run that the largest part of the print makes contact with the glass plate. The individual values, means, and SEM are presented. Horizontal lines show the statistically significant differences between analyzed groups.

**Figure 3:**
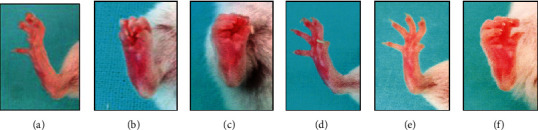
The effect of long-term injection of CII and LPS on the right hind paw. The images of right hind paws of selected animals taken after six times of injections of PBS (a), CII alone (b), a mixture of CII and KO3 LPS (c), YeO3 S LPS (d), YeO3 Ra LPS (e), and YeO3 Rd1 LPS (f).

Gait parameters of freely moving mice were determined with the CatWalk gait analysis system twenty days after last immunization. The analysis of BOS (base of support), the stand, and the maximal contact of fore and hind paws showed that CII injection induced differences in analyzed parameters (see Figure 2). The CII treatment led to the increase of hind paw BOS (see Figure 2(b)), mean stand (see Figure 2(d)), and the decrease in maximal paw contact (see Figures 2(e) and 2(f)). The coinjection of LPS led to the increase in hind limb BOS. Although no significant differences depending on endotoxin chemotype were observed, KO3 LPS led to the highest hind limb BOS, stand, and stand index. A similar pattern of reaction was observed also for the front paws (see Figures 2(a), 2(c), and 2(e)). Interestingly, treatment of mice with CII+KO3 LPS caused the erythema of right hind paws. Close effect was observed in the case of mice injected with CII and *Yersinia* Rd1 LPS (see Figure 3). To summarize, the obtained results indicated that repeated injections of collagen type II or its coinjections with LPS of any chemotype induced pathological changes in hind paws expressed as disturbances of animals' gait; however, only the KO3 LPS coinjection seemed to potentiate the effect caused by CII. However, no arthritis lesions in the paraffin section of the knee and ankle joints were observed in all analyzed groups of animals (data not shown).

### 3.2. *Yersinia* LPS Inner Core Heptoses Are Targets for Murine MBLs

**Figure 4:**
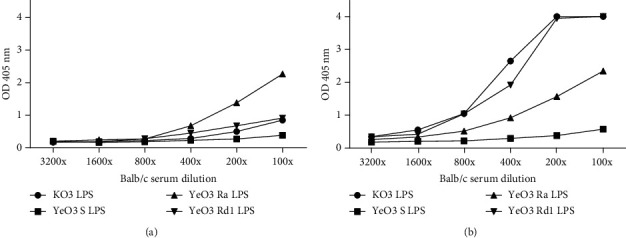
Interaction of murine mannose-binding lectins MBL-A (a) and MBL-C (b) with tested LPS.

To assess the possible influence of complement activation *via* the lectin pathway on *Yersinia* LPS adjuvant properties, first the interaction of mouse serum MBL-A and MBL-C with various LPS was analyzed in ELISA (see Figures 4(a) and 4(b)). *K*. *pneumoniae* O:3 LPS was used as the positive control. The obtained results indicated the differences in MBL specificity. MBL-C showed the strongest and similar affinity to KO3 LPS and YeO3 Rd1 LPS whereas the interaction of MBL-A with these LPS was much weaker. The binding of MBL-A and MBL-C to YeO3 Ra LPS was similar, although it should be remembered that the MBL-C level in the murine serum is several times higher compared to that of MBL-A. None of the murine MBL forms was able to interact with YeO3 S chemotype LPS. The obtained results indicated that rodent MBL-A and MBL-C (similarly to human MBL) recognize inner core heptoses within *Y*. *enterocolitica* O:3 LPS.

### 3.3. *Yersinia* LPS Inner Core Heptoses Are Responsible for Activation of Complement via the Lectin Pathway in Murine Serum

**Figure 5:**
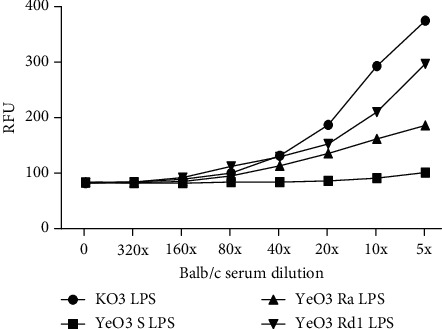
Activation of murine MASP-1 serine protease by tested LPS.

To test whether MBL binding to tested LPS leads to the activation of MBL-associated serine proteases (responsible for initiation of complement cascade), the ability of MASP-1 complexed with MBL to digest VPR substrate was analyzed (see Figure 5). Interaction of MBL-MASP complexes with KO3 LPS and YeO3 Rd1 LPS led to the activation of MASP-1 at the highest level. No fluorescence increase was observed for YeO3 S chemotype (strain 6471/76-c) LPS.

The obtained results indicate that YeO3 Rd1 mutant LPS (consisting of lipid A and heptoses-containing inner core) has a similar ability to activate MBL-MASP-1 complex as the mannose-rich KO3 LPS.

### 3.4. *Yersinia* Lipopolysaccharides Are Immunogenic but Have No Greater Adjuvant Activity

**Figure 6:**
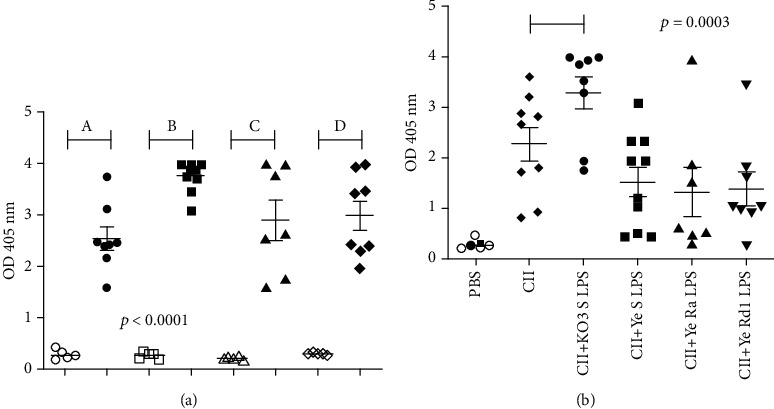
Immunogenicity (a) and adjuvant properties (b) of tested LPS. (a) The antibodies against KO3 LPS (A), YeO3 S LPS (B), Ye Ra LPS (C), and Ye Rd1 LPS (D) were tested in sera (1 : 5) of mice injected with PBS (open symbols) or corresponding LPS (closed symbols). (b) The antibodies against type II collagen were tested in sera (1 : 200) obtained from animals treated with PBS only, CII alone, or the mixture of CII and tested LPS; the antibodies against CII were detected. The results were analyzed with an ANOVA Kruskal-Wallis with Dunn's post hoc test. The individual values, means, and SEM are presented. Horizontal lines show the statistically significant differences between analyzed groups.

Immunization of animals with the mixture of CII and LPS obviously led to the production of both anticollagen and antiendotoxin antibodies (see Figures 6(a) and 6(b)). No antibodies were detected against collagen and tested LPS in sera of mice treated with PBS only. Among tested endotoxins, KO3 LPS only had an adjuvant activity, enhancing anti-CII antibody synthesis. In contrast, *Yersinia* LPS rather decreased (although not markedly) the production of collagen type II-specific antibodies.

**Figure 7:**
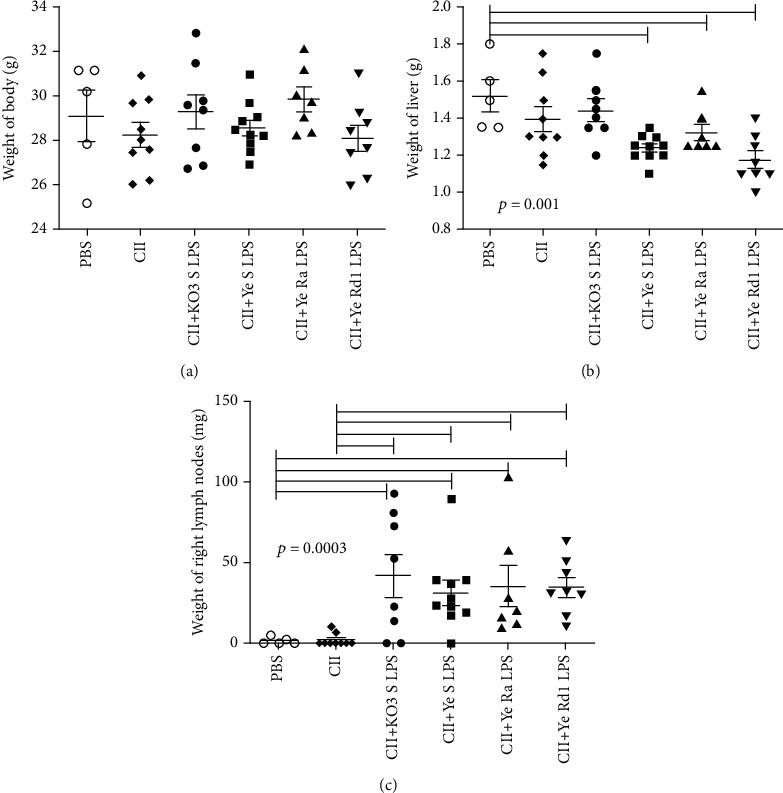
The consequences of long-term treatment of mice with collagen type II and its mixture with LPS. Body weight (a), weights of the liver (b), and inguinal right lymph nodes (c) were analyzed using ANOVA Kruskal-Wallis with Dunn's post hoc test. The individual values, mean, and SEM are presented. Horizontal lines show the statistically significant differences between analyzed groups.

The long-term treatment of animals with CII or CII plus LPS did not influence significantly their body weight (see Figure 7(a)). However, injection of CII and *Yersinia* LPS, independently of its chemotype, caused a significant decrease in the weight of the liver in comparison to mice treated with PBS only (see Figure 7(b)). No hepatotoxicity was observed in the case of KO3 LPS. Moreover, the weight of livers of animals treated with Ye Rd1 chemotype LPS was lower than in the case with mice immunized with CII only. However, immunization with the mixture of CII and LPS (independently of its chemotype) was associated with significantly enlarged inguinal lymph nodes (see Figure 7(c)).

### 3.5. Long-Term *Yersinia* LPS Treatment Influences Hepatic MBL-C Expression

**Figure 8:**
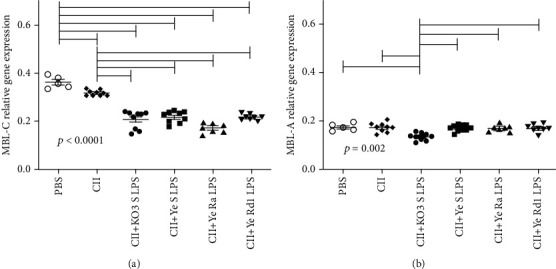
Influence of long-term immunization with CII and LPS on *Mbl2* (MBL-C) and *Mbl1* (MBL-A) gene expressions. The MBL-C (a) and MBL-A (b) relative gene expressions were determined in the livers of immunized mice. The results were analyzed with ANOVA Kruskal-Wallis with Dunn's post hoc test. The individual values, mean, and SEM are presented. Horizontal lines show the statistically significant differences between analyzed groups.

To test the influence of long-term immunization with collagen and *Yersinia* LPS (differing in the length of polysaccharide chain) on MBL-A and MBL-C expressions in the liver, the real-time PCR with the use of specific primers was performed. KO3 LPS was used as the positive control while PBS alone was used as the negative control. A significant decrease of MBL-C expression after CII treatment in comparison with the negative control was found (see Figure 8(a)). Moreover, the coinjection of LPS was associated with a further drop of MBL-C expression. In contrast, with an exception for KO3 LPS, no greater differences were found for MBL-A (see Figure 8(b)).

Similarly, the administration of CII or its mixture with tested *Yersinia* LPS was associated with the significant lowering of MBL-C (protein) serum level (data not shown). In contrast, the CII and KO3 LPS injection induced that in 25% (2/8) mice only. The trend towards lower MBL-A serum concentration was observed after usage of CII alone or when CII was accompanied with *Yersinia* LPS. The borderline statistical significance was however reached in the case of Ra and Rd1 LPS only.

## 4. Discussion

Collagen-induced arthritis (CIA) in rats or mice is a common animal model of inflammatory arthritis that resembles the human rheumatoid arthritis (RA) [38]. RA is a chronic autoimmune disease with systemic clinical manifestation, but first of all, it is associated with inflammation of joints, which can lead to joint damage and disabilities. Symptoms include pain in affected joints, limitation of motion, and functional impairment and are associated with swelling, warmth, and tenderness in affected joints [39]. Extensive inflammation and pain may lead to disuse of the affected limb. The *Yersinia* gastrointestinal infection may be associated with development of another type of arthropathy, reactive arthritis, and *Yersinia-*derived structures were detected in the affected joints. Furthermore, *Yersinia-*reactive antibodies were found relatively common among patients suffering from juvenile idiopathic arthritis [25]. Taking into account the involvement of known arthritogenic implications of infections with those bacteria as well as adjuvancity and proinflammatory activity of endotoxin, we hypothesized that *Yersinia* LPS may contribute to the pathogenesis of collagen-induced arthritis. In order to determine whether *Yersinia* LPS may influence the locomotion of mice in collagen induced arthritis (CIA) model, we used CatWalk gait analysis system (see Figure 2). Since *Yersinia* LPS was suggested to be processed by phagocytic cells in joints [8], beside LPS of S chemotype, LPS representing Ra and Rd1 chemotypes were also included in the experiments. The long-term treatment of mice with the mixture of collagen and *Yersinia* LPS (but not collagen alone) was associated with significantly increased BOS limbs in comparison with the control mice. Moreover, the mean hind limb maximal contact was markedly decreased in CII+LPS-treated animals in comparison with the reference group. No differences between *Y*. *enterocolitica* LPS representing wild-type Ra or Rd1 chemotypes were observed. It may suggest that this LPS activity is associated with inner core-lipid A region, present in all analyzed LPS preparations. It should be noticed, however, that a similar effect (although less pronounced) was observed when collagen alone was injected. Notwithstanding, the strongest influence on mice motility was observed in the case of CII mixed with KO3 LPS, with the previously evidenced strong adjuvant potency [17]. Although no visible swelling was shown, paws of some animals were red and very sensitive to touch (see Figure 3). However, no pathological signs of inflammation in hematoxylin/eosin-stained sections of paraffin-embedded knee and ankle joints were observed (data not shown). No difference in the body weight among analyzed groups was noticed, although *Yersinia* LPS (independently of chemotype) seemed to be hepatotoxic (see Figure 7).

Collagen type II is one of the major constituents of the articular cartilage matrix proteins. Antibodies to native type II collagen have been reported in a number of diseases, including rheumatoid arthritis [40]. They were found in the sera of 10% of RA patients [41]. They are also present in the synovial fluid [42]. Moreover, administration of exogenous collagen induced antibodies reacting to animal's own collagen. Treatment of mice with a cocktail of anti-CII monoclonal antibodies induces arthritis (CAIA-model) [11].

Injection of collagen in the presence of complete Freund's adjuvant increased the arthritis incidence. However, the LPS of Gram-negative bacteria is also able to act as adjuvants. LPS accelerated the disease onset and exacerbated the clinical score in the CIA model [43]. Moreover, mice are often treated with LPS in collagen antibody-induced arthritis (CAIA) to synchronize the time of onset. We analyzed whether *Yersinia* LPS may contribute to the production of anti-collagen type II antibodies (or immune response) with arthritogenic potential. However, although all tested LPS were able to induce specific anti-LPS antibodies, only CII+KO3 LPS and CII alone stimulated the production of antibodies reacting with collagen at a high level. *Yersinia* LPS (independently of the chemotype) seem to decline the induction of the anticollagen antibodies. In contrast, treatment of animals with the mixture of CII and LPS (independently of the LPS chemotype and origin), but not with CII alone, was associated with a significant enlargement of the right inguinal lymph nodes (see Figure 7(c)). The obtained results suggest that the *Y*. *enterocolitica* O:3 LPS does not contribute to the development of arthritis through the induction of the production of anticollagen antibodies. Similarly, in the CIA model, injection of LPS was not associated with significantly increased level of anti-CII antibodies in mice, in comparison with saline, but rather with increased expression of genes encoding for inflammatory mediators in arthritic paws [44].

Numerous studies have demonstrated that the complement system plays a significant role in the induction of the inflammation essential for arthritis development. Complement factors and their activation products were detected in synovial fluids [25, 45]. The deposition of products of complement activation on articular collagenous tissues was reported by Cooke et al. [46]. Moreover, inhibition of complement was found to be associated with less severe disease [47], and mice deficient in complement components occurred resistant to arthritis [48, 49]. The crucial role of the complement alternative pathway (AP) in the induction of arthritis associated with anticollagen antibodies was demonstrated by Banda et al. [50], and *N*-glycans of IgG in immunocomplexes were shown to induce AP activation in the CAIA model [51]. Although classical (CP) and lectin (LP) routes of complement activation alone seem not to be essential in the pathogenesis of arthritis, there is a link between the AP and LP. MASP-3 and MASP-2 serine proteases of LP were shown to contribute to AP activation and disease development. Banda et al. [27] demonstrated the key role of MASP-3 (unbound or associated with ficolin-B but not with MBL-A or MBL-C) in the cleavage of profactor D, and MASP-1/3-deficient mice were protected from arthritis development [38, 52], whereas disease activity was decreased in MASP-2^−/−^/sMAp^−/−^ animals [26].

Our results indicate that long-term treatment of animals with the mixture of CII and LPS was associated with significant mobility impairment of tested animals. This may result from inflammation induced by the LPS polysaccharide-dependent activation of the complement alternative pathway, boosted by LPS-induced increased expression of factor D [28]. Moreover, LPS-induced hepatotoxicity may lead to decreased expression of MBL-C/MBL-A. It may be associated with an increase of free MASP-3 in serum, able to cleave profactor D and to accelerate AP activation, contributing to arthritis development. In this work, of the tested LPS, the strongest activity was observed for KO3 LPS. This endotoxin was shown to bind human MBL *via* O-specific polysaccharide chains and *via* heptoses present in the LPS inner and outer cores [19, 53]. A similar but weaker activity was demonstrated for *Yersinia* Rd1 LPS (lacking OPS), which was able also to bind human MBL [25]. Both LPS were also shown to potently bind mouse serum MBL-C (see Figure 4) and to activate LP (see Figure 5).

Interestingly, KO3 LPS only was shown to enhance significantly the collagen type II-specific antibody production. Although long-term treatment of animals with all tested LPS led to decreased expression of MBL-C-specific mRNA, only KO3 LPS caused also the significant drop in *Mbl1* (MBL-A) mRNA. Interestingly, Ruseva et al. [54] demonstrated that MBL-A/C deficiency may be associated with stronger immune response, since it was found that MBL-A^−/−^/MBL-C^−/−^ knockout mice showed significantly higher production of antibodies against hepatitis B virus surface antigen than wild-type mice. That however does not explain the weakened anti-CII response (lower level of specific antibodies) in *Yersinia* LPS-treated animals.

## 5. Conclusions

To conclude, our data suggest that the arthritogenic activity of *Yersinia enterocolitica* O:3 lipopolysaccharide is very likely not associated with its ability to boost anti-collagen II Ab production. However, its interaction with MBL-C and subsequent MASP activation, accompanied with influence on corresponding *Mbl2* gene expression, may contribute to some adverse effects that might be related to cross-talk with the complement alternative pathway (*via* pro-D activation) involved in arthritis development.

## Figures and Tables

**Table 1:** Description of bacterial strains used in this study.

Strain	Description	LPS chemotype	MBL target	References
*K*. *pneumoniae*	Serotype O:3 (O:3 K55^−^ strain 5505*Δ*cps) OPS is a homopolymer of mannose	S type LPS (O-antigen+complete core) S (LA-IC-OC-OPS)	OPS, outer and inner core heptoses	[19, 31]
*Y*. *enterocolitica* 6471/76-c	Ye serotype O:3, patient stool isolate, wild-type strain; cured of virulence plasmid (pYV^−^)	S type LPS (O-antigen+complete core) S (LA-IC-OPS) and Ra (LA-IC-OC)	Inner core heptoses	[20, 24]
*Y*. *enterocolitica* YeO3-c-R1	Spontaneous rough derivate of strain 6471/76-c	Ra type LPS with complete core Ra (LA-IC-OC)	Inner core heptoses	[21, 24]
*Y*. *enterocolitica* YeO3-c-R1-M181	YeO3-c-R1 (*galU*::Cat-Mu, clmR) Deep rough mutant of strain YeO3-R1, pYV-negative, chloramphenicol resistant. Insertion of Cat-Mu fragment (1, 2 kb) in *galU* gene in the LPS inner core biosynthesis gene cluster	Deep rough type LPS with the truncated inner core (the outer core missing) Rd1 (LA-4/8 IC)	Inner core heptoses	[23, 24]

## Data Availability

Data are available on request from corresponding author.
